# Adipose-derived stem cell-mediated paclitaxel delivery inhibits breast cancer growth

**DOI:** 10.1371/journal.pone.0203426

**Published:** 2018-09-07

**Authors:** Maria Giovanna Scioli, Simona Artuso, Carmen D'Angelo, Manuela Porru, Federico D’Amico, Alessandra Bielli, Pietro Gentile, Valerio Cervelli, Carlo Leonetti, Augusto Orlandi

**Affiliations:** 1 Anatomic Pathology, Department of Biomedicine and Prevention, Tor Vergata University, Rome, Italy; 2 SAFU, IRCCS-Regina Elena National Cancer Institute, Rome, Italy; 3 Unit of Oncogenomics and Epigenetics, IRCCS-Regina Elena National Cancer Institute, Rome, Italy; 4 Plastic and Reconstructive Surgery, Department of Biomedicine and Prevention, Tor Vergata University, Rome, Italy; 5 Anatomic Pathology, Department of Biomedical Sciences, Catholic University Our lady of Good Counsel, Tirana, Albania; Università degli Studi della Campania, ITALY

## Abstract

Breast cancer represents the main malignancy in women and autologous fat grafting is a diffuse procedure in the management of post-surgical breast defects causing patients’ psychosocial problems, with high costs for the public health. Recently, beneficial effects of fat grafting during post-surgical breast reconstruction have been amplified from the enrichment with human adipose-derived stem cells (ASCs) present in the stromal vascular fraction (SVF) of adult adipose tissue isolated during intraoperatory procedures. The major concern about the ASC enrichment during post-surgery breast reconstruction depends on their potential ability to release growth factors and hormones that can promote proliferation of residual or quiescent cancer cells, with the risk of *de novo* cancer development or recurrence. The recent description that adult stem cells primed *in vitro* may be vehicle for anti-cancer drug delivery offers a new vision concerning the role of ASCs in breast reconstruction after cancer surgery. Paclitaxel (PTX) is a chemotherapeutic agent acting as a microtubule-stabilizing drug inhibiting cancer cell mitotic activity. We optimized PTX loading and release in cultured ASCs and then analyzed the effects of PTX-loaded ASCs and their conditioned medium on CG5 breast cancer survival, proliferation and apoptosis *in vitro*, and inCG5 xenograft *in vivo*. We documented that ASCs can uptake and release PTX *in vitro*, with slight cytotoxic effects. Interestingly, PTX-loaded ASCs in co-culture, as well as conditioned medium alone, inhibited CG5 cell proliferation and survival *in vitro* and xenograft tumor growth *in vivo*. The antitumor effect of PTX-loaded ASCs may offer a new perspective concerning the use of ASCs during breast reconstruction becoming an additional local preventive chemotherapeutic agent against tumor recurrence. However, further experiments *in vitro* and *in vivo* are needed to collect more evidence confirming the efficacy and safety in cancer patients.

## Introduction

Breast cancer treatment is currently focused on patients’ cure rate and maintenance of the quality of life [1]. Collaboration between breast and plastic surgeons helped to decrease the disabling effects of breast surgical mutilation. Autologous fat grafting is widely used during breast reconstruction after cancer surgery [2,3]. Adult adipose tissue contains different cell types, such as adipocytes, smooth muscle cells, macrophages, and pericytes [4–6]. Perivascular stromal cells have the potential to form *in vitro* and *in vivo* bone, cartilage and fat tissue, as well as skeletal muscle [7–9] and are named multipotent adipose-derived stem cells (ASCs). The latter can be easily isolated from subcutaneous adult adipose tissue after liposuction by enzymatic digestion and culture of the stromal vascular fraction (SVF) [7,10,11]. ASCs share some similarities with bone marrow-derived mesenchymal stem cells (MSCs) [12]. Unfortunately, the long-term beneficial effects of fat grafting are limited, with a rate from 25% to 80% [13]. Recently, the ASCs/SVF enrichment of autologous fat grafting for regenerative surgery reported positive results in wound healing and fat graft maintenance after post-surgical breast reconstruction, strongly supporting its use in the routinely clinical practice [14,15]. Nevertheless, the injection of stem cells during tissue reconstruction procedures has raised a question regarding the safety of these procedures in cancer patients [16]. There is no doubt that MSCs can contribute to tumor development and progression, by promoting neoangiogenesis and invasion [17]. The possibility that breast cancer cells might still be present in the residual mammary parenchyma after conservative surgery cannot be completely ruled out [18]. Consequently, the injection of stem cells in these areas might stimulate the proliferation of ‘dormant’ breast cancer cells. In fact, the relationship between ASCs and breast epithelial cells remains unclear. Preliminary data documented an active and mutual connection between the epithelial and stromal component in the progression of breast cancer [19,20]. Moreover, the receptor pathways regulating ASC proliferation and differentiation are also involved in breast cancer biology. ErbB tyrosine kinase receptor (ErbB) families are reported to modulate cancer stem cell growth and differentiation [21–23]. Recently, some authors highlighted the presence of EGFR and ErbB2 expression in ASCs [24]. In addition, estrogen stimulates breast cancer cell proliferation by the transcription of different growth factors [25,26]. In contrast, pre-clinical experiments seem to support that ASCs may favor the peritumoral desmoplastic reaction by extracellular matrix deposition and neoangiogenesis [27]. In *vivo* studies demonstrated that ASCs interact differently on active or quiescent breast cancer cells. The latter are rather independent and proliferate slowly [28,29]. To date, clinical trials and follow-up studies are not clear about the increased risk of cancer recurrence or new onset after lipofilling [13, 30–35]. In a study focused on patients previously diagnosed with breast intraepithelial neoplasia, the lipofilling group did not display a significant higher risk of local recurrence when compared to the untreated group [31]. However, in a study of 37 cases, the lipofilling patients showed higher risk of local relapse close to the lipofilling injection when the analysis was limited to breast intraepithelial neoplasia [32]. From a scenario of conflicting opinions, the potential role of ASCs in cancer progression is still debated [33]. Conventional breast cancer therapies include surgery, chemotherapy and radiotherapy. Recent preclinical studies based on MSC ability to home in the tumor microenvironment, suggested their use as candidates to delivery anti-cancer drugs [37–40]. Paclitaxel (PTX) is a widely used chemotherapic drug that acts as microtubule-stabilizing agent, inhibiting cancer cell mitosis [41]. It has been reported that MSCs and ASCs can uptake and release PTX in vitro, so inhibiting the proliferation of some cancer cell lines [39,42–44], but poor information about breast cancer [45]), especially concerning ASC delivery, is available. In the present study, we investigated the antitumor effects of PTX-loaded ASCs on human CG5 breast cancer cell line and in mouse xenografts. We speculated to take advantage of the ability ASCs to migrate into the microenvironment of potential residual or quiescent breast cancer cells during post-surgical breast reconstruction. Their capacity to locally vehicle and release drugs can be hypothesize as a potential new and easily accessible tool for an adjuvant anti-cancer activity, so reducing the risk of local cancer recurrence but maintaining their beneficial regenerative properties.

## Materials and methods

### Ethics statement

Investigation was performed in accordance with the ethical standards, according to the Declaration of Helsinki, and to national and international guidelines, and the study has been approved by the authors’ institutional review board (Tor Vergata Ethical Committee). Lipoaspirate samples were obtained during elective surgery with the patient’s informed, written consent and in accordance with the guidelines of the Tor Vergata Committee on Human Research. Animal protocols were approved by the Italian Ministry of Health; Department of Public Health, Animal Health, Nutrition and Food Safety. All procedures were performed under anesthesia, and all efforts were made for minimize potential pain, suffering, or distress, and enhance animal welfare, according to the national and international directives (D.L. March 4, 2014, no. 26; directive 2010/63/EU of the European Parliament and of the council; Guide for the Care and Use of Laboratory Animals, United States National Research Council, 2011).

### Fat collection, ASC isolation and expansion

Subcutaneous adipose tissue was harvested during liposuction procedures. The SVF was obtained by enzymatic digestion of fat samples and centrifugation, as reported [10]. The SVF pellet was resuspended in few microliters of growth medium and passed through a 100-μm Falcon strainer (Becton and Dickinson, Sunnyvale, CA). The suspension was plated, and ASCs cultured in complete medium according to our previous study [10]. For all experiments cells were used between passage 1 and 3.

### PTX-uptake and release by ASCs

PTX was purchased from Adipogen (Vinci-Biochem, Italy). Priming of ASCs was performed as previously described [46]. Briefly, ASCs were incubated for 24h with different concentrations of PTX (from 10nM to 10μM). The activity of PTX on ASCs was determined by a cytotoxicity test (MTT assay) and flow cytometric analysis (see below). Incorporation and subsequently release of PTX from ASCs was assessed by HPLC analysis, using a standard sample of PTX in PBS. The latter showed a peak of identical retention time of PTX eluted from the PTX-loaded ASC-conditioned medium [46].

### Immunophenotype

PTX-loaded and control ASCs (at passage 3) were resuspended in PBS for analysis and assessed for size by using forward and side light-scatter measurements. Flow cytometric analysis was performed [24] using BD FACSCanto II (BD Biosciences, CA, USA). For immunophenotype, cells were incubated with the following fluorochrome-conjugated mouse monoclonal antibodies: CD44-FITC (Abcam; Cambridge, UK), CD90-PE (Abcam), CD34-FITC (BD Biosciences), CD73-FITC (eBioscience, Thermo Fisher Scientific, MA, USA) and CD105-PE (eBioscience) as recommended by manufacturer. Appropriate isotype controls are used as negative controls.

### Cell cycle and apoptosis analysis

For cell cycle analysis, cells were washed in PBS, fixed in 70% ethanol at 4°C for 30 min and resuspended in PBS containing 0.01% RNase (Sigma-Aldrich). Then, cells were incubated with 25μg/ml propidium iodide and 2% Triton-X 100 (Sigma-Aldrich) at room temperature for 15 min. Flow cytometry was used to analyze the percentage of cells in different phases of the cell cycle. For each analysis, 30,000 events were collected. Apoptosis was analyzed by annexin V assay. Annexin V-FITC versus PI assay (FITC Annexin V/ Dead Cell Apoptosis Kit, V-13242, Molecular Probes, Invitrogen). Briefly, adherent cells were harvested, suspended in the annexin-binding buffer (1 x 10^6^cells/ml) and incubated with the annexin V-FITC and propidium iodide for 15 min, at room temperature in the dark, then immediately analyzed by flow-cytometry. The data are presented as bi-parametric dot plots showing the annexin V-FITC green fluorescence versus the PI red fluorescence. Cell cycle distribution and percentage of apoptotic cells were analyzed using FACSDiva Software (BDIS) and ModFit LT (Verity Software House, Topsham, ME). Flow cytometric analysis was performed [21] using BD FACSCanto II, FACSCalibur or FACSCelesta^TM^ (BD Biosciences, CA, USA).

### Cytotoxic assay (MTT assay)

Firstly, cytotoxic activity of PTX was assessed on ASCs and CG5 breast cancer cells. Cells (15x10^3^) were cultured in triplicate in complete medium in flat bottom 96 well plates in the presence of different PTX concentrations (10nM-10μM) for 24 hours, before serum starvation for 16 hours. Cytotoxic activity was also assessed on CG5 incubated with conditioned medium from PTX-loaded ASCs (Table 1). Briefly, ASCs were incubated with PTX (10nM-10μM) for 1, 6, 12 and 24 hours. To obtain conditioned media, ASCs cells were treated or not with PTX at different doses for 6 hours. Then medium was removed and replaced with completed medium. After 24, 48 or 72 hours conditioned media containing PTX released from ASCs were collected and added to CG5, ASCs or co-culture CG5/ASCs. After 12, 24 and 48 hours conditioned media were collected and added to CG5 cells for 7 days. Control cells were cultured only with standard medium or with conditioned medium of unloaded ASCs. Cell viability was measured by MTT assay, according to the manufacturer recommendations (Sigma-Aldrich). Absorbance as optical density units was determined at 570 nm by a microplate reader (Sunrise TECAN, LabX, Midland, ON, Canada). The inhibitory concentration (IC_50_) were determined according to the Reed and Muench formula [46].

**Table 1:** Cytotoxic activity of PTX-loaded ASC conditioned medium on CG5 human breast cancer cells (MTT assay).

	1h of PTX loading (I.C.50) **	6h of PTX loading (I.C.50)	12h of PTX loading (I.C.50)	24h of PTX loading (I.C.50)
12h*	0,34 μM	0,15 μM	0,013 μM	0,059 μM
**24h**	>0,4 μM	0,029 μM	= 0,4 μM	0,3 μM
**48h**	>0,4 μM	>0,4 μM	>0,4 μM	>0,4 μM

* Conditioned medium of ASCs after 12, 24 or 48h from PTX loading (10nM-10μM PTX for 1, 6, 12 and 24 hours).

** IC50 calculated on CG5 after 7 days of treatment with PTX-loaded ASC conditioned medium.

### Clonogenic assay

ASCs were loaded or not with different concentrations of PTX (100nM-10μM) for 6 hours. Then, ASCs were mixed with CG5 with a ratio 1:2 (250:500 cells per 6-cm dish), plated, and kept in a humidified incubator at 37°C/ 5% CO2. The CG5/ASC co-cultures were also treated with PTX-loaded ASC conditioned medium previously obtained. The count of colonies was performed after 11 days and compared with CG5 alone or CG5 treated with PTX free (3nM). Colonies were fixed and stained with 1% methylene blue in 0,1% methanol and percentage calculated. In addition, in some experiences, after 24 hours of treatment, cells were processed for Annexin V/PI analysis. Experiments were carried out in triplicate.

### Tumor xenografts

CD-1 female nude (nu/nu) mice, 4–6 weeks old and weighing 22–24 g, were purchased from Charles River Laboratories (Calco, Italy). A preliminary study was performed on nude mice (5 mice/group) injected intramuscularly (i.m.) with CG5 cells alone (3x10^6^) or mixed, with a ratio of 2:1, with ASCs (1.5x10^6^) unloaded or primed with PTX (2μM) for 6 hours, and the incidence and growth of tumors were followed. Based on the preliminary results obtained, a second experiment was performed and nude mice (5 mice/group) injected i.m. with CG5 cells (1x10^6^) alone or mixed, at ratio of 1:1, with ASCs (1x10^6^) unloaded or primed with PTX (2μM or 4μM) for 6 hours, trypsinized and co-injected with CG5 cells. Another control group was CG5 cells (1x10^6^) mixed with PTX free (4μM). Tumor sizes were measured three times a week in two dimensions by a caliper and tumor weight was calculated using the following formula: a×b^2^/2, where a and b are the long and short diameter of the tumor, respectively. Animals received sterile food and water. Animals were monitored twice daily with health monitor forms and managed aseptically. None of mice showed sign of illness or died before the experimental endpoint in this study. Mice were euthanized with CO_2_and tumors removed, fixed in 10% neutral buffered formalin and paraffin-embedded for histology.

### Microscopic and morphometric analysis

Haematoxylin&Eosin (H-E)-stained sections of tumor xenografts were visualized using E600 Eclipse microscope (Nikon, Tokio, Japan) and images captured by Nikon Digital camera (DXM1200F) using the ACT-1 software (Nikon). For morphometric analysis, slides were scanned by using BioImagene iScan™ at 2x magnification (Roche Diagnostics, Monza, Italy) and analyzed by Scion Image software (Scion Corp., Frederick, MD). The total tumor area (mm^2^), necrotic area (light pink eosinophilic area inside tumor mass, mm^2^), residual tumor area [tumor area-necrotic area] and the percentage of necrosis [(necrotic area/ total tumor area) x100] were measured.

### Statistical analysis

Results were expressed as the mean of at least three different experiments plus standard error (SEM) and analyzed by Student’s *t*-test. Differences were considered statistically significant for *P*< 0.05.

## Results

### The effect of PTX uptake on ASC behavior

**Fig 1:**
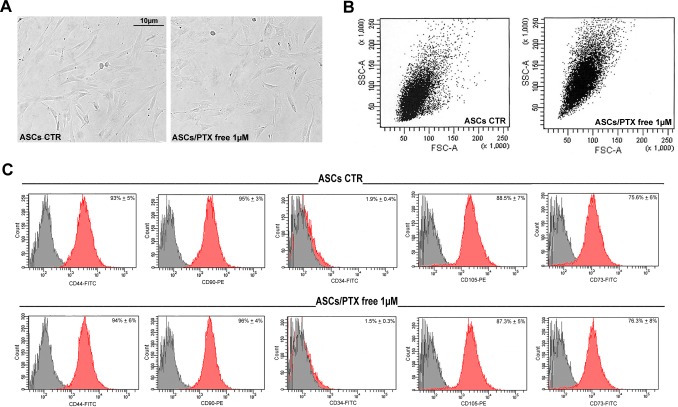
Morphology and stem immunophenotype of PTX treated ASCs. A) Representative images of cultured ASCs treated with PTX 1μM for 6 hours. B) Flow cytometric analysis of side and forward scatter of control (CTR) and PTX-loaded ASCs (ASCs/PTX; 1μM for 6 hours) showing an increased intracytoplasmic granularity of PTX-loaded ASCs. C) Histograms showing similar percentages of CD90, CD44, CD34, CD105 and CD73 positive cells in CTR (untreated) and PTX treated ASCs. The grey histograms indicate isotype controls, while the red histograms antibody expression.

Preliminary experiments of PTX loading and releasing demonstrated that ASCs can uptake and release PTX [41]. PTX (1μM)-primed ASCs were like unloaded control ASCs (at passage 3) in terms of cell morphology (Fig 1A). However, flow cytometry analysis of PTX-primed ASC side scatter showed an increased intracytoplasmic granularity, in line with PTX incorporation (Fig 1B). In addition, stem immunophenotype (Fig 1C) did not revealed any significant changes (CD90, CD44, CD34, CD73 and CD105 expression).

**Fig 2:**
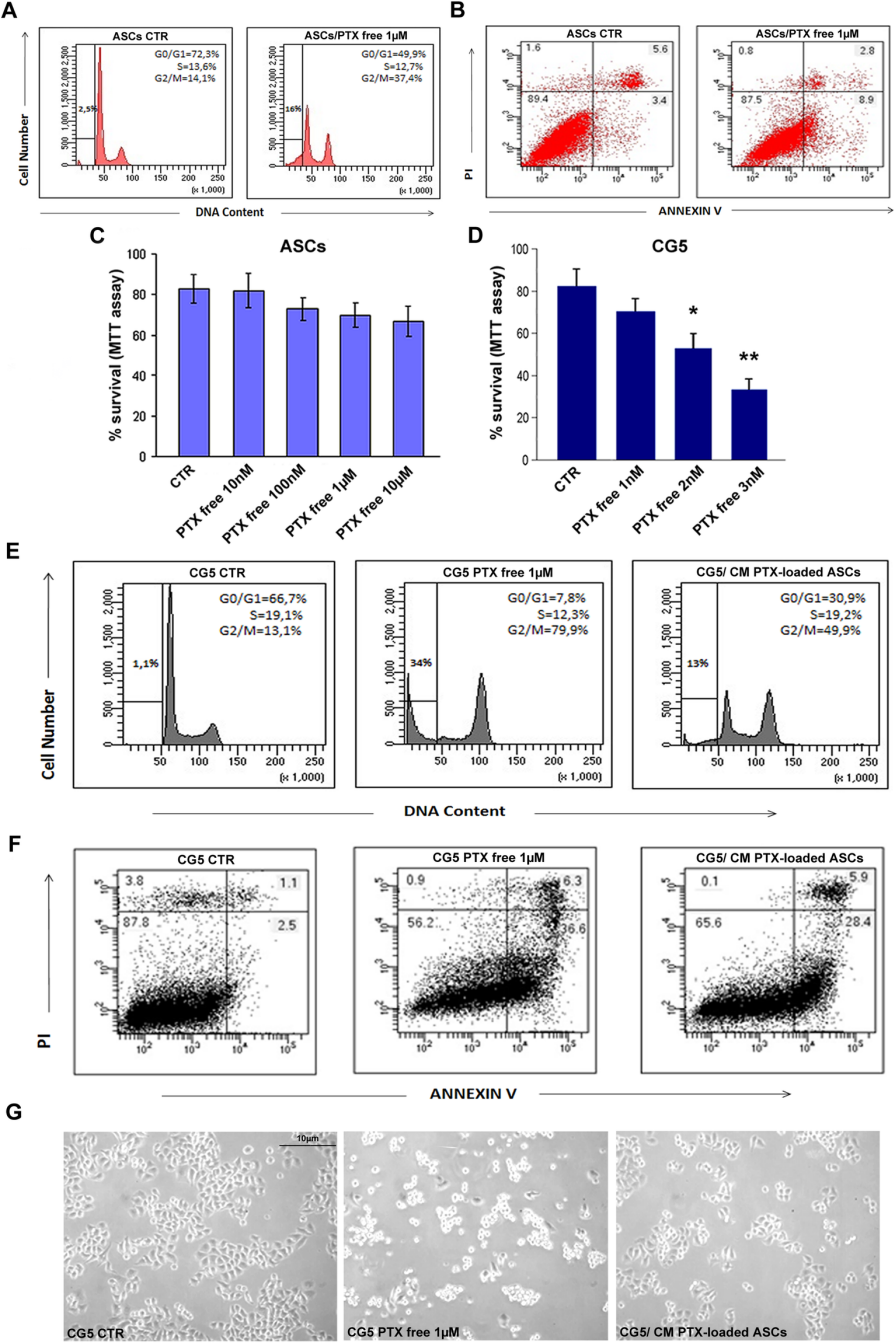
Cell cycle, survival and apoptosis of PTX treated ASCs and CG5. A) Cell cycle analysis by flow cytometry of CTR (untreated) and PTX treated ASCs (1μM for 6 hours). Adherent and not adherent cells were processed for PI staining to evaluate cell cycle. B) Cytofluorimetric bi-parametric analysis of the annexin V versus PI staining assay. The percentage reported in the annexin V^+^/PI^+^ region of each histogram represents the apoptotic cells. C, D) MTT assay of ASCs and CG5 treated with different concentrations of PTX for 6 hours, respectively. E) CG5 cells were treated with PTX free at 1 μM or with conditioned medium previously obtained by PTX primed ASCs at 1 μM. Adherent and not adherent cells were processed for cell cycle analysis. The percentages of cells in the different phases of cell cycle were reported inside the relative histograms. F) Cytofluorimetric bi-parametric analysis of the annexin V versus PI staining assay. G) Representative images of CG5 cell cultures with different treatments. Abbreviation: CM, conditioned medium.*t*-test: * and ** indicate p< 0.01 and p< 0.001, respectively.

Cell cycle analysis (propidium iodide staining, Fig 2A) of ASC total population (adherent and not-adherent cells) revealed that although PTX at high concentrations (free or conditioned medium) induced an increase of sub G0/G1 population (16% vs 2.5% CTR) and a moderate G2/M phase arrest (37.4% vs 14% CTR), no significant effect of cytotoxicity in terms of apoptosis (3.4% vs 8.9% CTR) or necrosis was observed, as shown by Annexin V/PI staining (Fig 2B and Panel A in S1 Fig). In fact, by means of the annexin V *versus* PI staining assay, ASCs showed after treatment with PTX free (Fig 2B) or with their conditioned medium (Panel B in S1 Fig), respectively 8.9% and 10,8% of apoptosis, demonstrating a negligible effect of PTX in these cells. This finding was also confirmed by the high percentage of PTX treated ASC survival by MTT assay (Fig 2C).

### Co-culture of PTX-loaded ASCs and conditioned medium inhibits proliferation and survival of CG5 breast cancer cells

As reported in Fig 2D, MTT assay documented a strong sensitivity of CG5 breast cancer cells to PTX free, already starting from 2nM concentration, with a significant reduction of survival (2nM and 3nM, p< 0.01 and p< 0.001, respectively). Cell cycle analysis (Fig 2E) of CG5 total population revealed that PTX treatment caused a strong arrest in G2/M phase (79.9% vs 13,1% CTR) with a manifest reduction of G0/G1 population (7.8% vs 66.7% CTR) and increase of sub G0/G1 phase (34% vs 1.1% CTR). This finding was confirmed by cell cycle analysis of CG5 cells treated with PTX-loaded ASC conditioned medium (Fig 2E) that showed a significant arrest in G2/M phase of CG5 cells (49.9% vs 13,1% CTR), a reduction of G0/G1 population (30.9% vs 66.7% CTR) and increase of sub G0/G1 phase (13% vs 1.1% CTR). Cytotoxicity analysis by Annexin V/PI staining (Fig 2F) revealed that CG5 cells died for apoptosis around 36.6% of the population after treatment with PTX free and 28.4% after treatment with PTX-loaded ASC conditioned medium, indicating the high toxicity of conditioned medium comparable to free drug in CG5 cancer cells. No apoptosis was observed in either of the control line (Fig 2F). Representative images of these treatments were also showed (Fig 2G).

**Fig 3:**
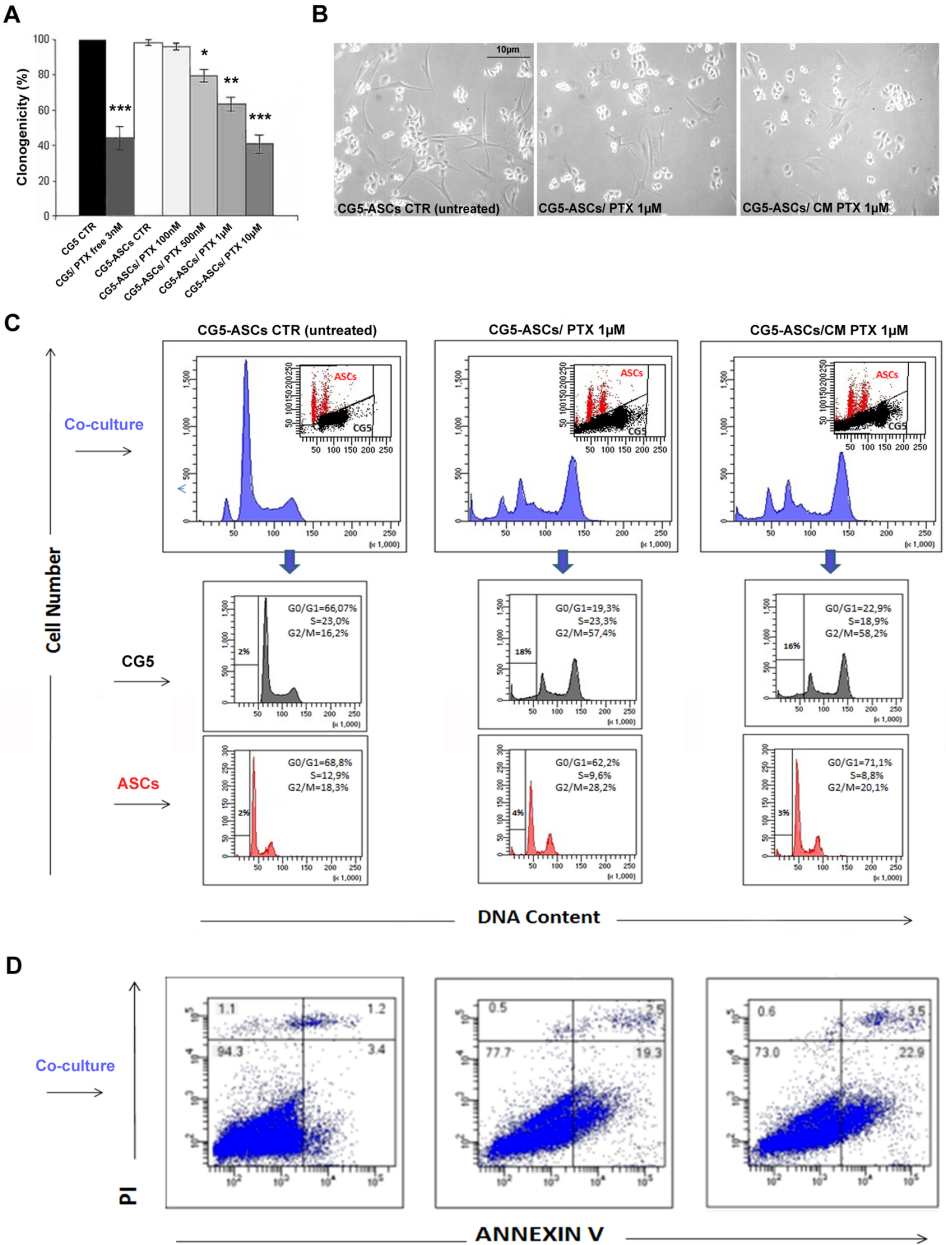
Clonogenicity, cell cycle and apoptosis of CG5/ASC co-cultures in the presence of PTX. A) The inhibitory activity of PTX-loaded ASCs on CG5 proliferation by clonogenic assay. ASCs were primed or not with different concentrations of PTX (for 6 hours), mixed with CG5 and the number of colonies evaluated compared with CG5 alone or treated with PTX free (3nM). B) Representative images of CG5 cell cultures with different treatments. C) Cell cycle analysis by flow cytometry of CG5/ASC co-cultures with different treatments (untreated ASCs, PTX-loaded ASCs or CM PTX-loaded ASCs at 1μM). The two cell populations were separated by analytical sorter in bi-parametric analysis of DNA content versus forward scatter. D) Cytofluorimetric bi-parametric analysis of the annexin V versus PI staining assay. The percentage reported in the annexin V^+^/PI^+^ region of each histogram represents the apoptotic cells. Abbreviation: CM, conditioned medium.*t*-test: *, ** and *** indicate p< 0.05, p< 0.01 and p< 0.001, respectively.

Clonogenic assay with co-cultures (CG5 mixed with ASCs) documented a significant reduction in the clonogenic ability of CG5 with PTX-loaded ASCs, starting from 500nM concentration, in a dose-dependent manner (p< 0.05; Fig 3A and 3B). In addition, cell cycle analysis of the two cell populations showed an evident arrest in G2/M phase of CG5 cells with a high reduction of G0/G1 population and increase of sub G0/G1 cell population (Fig 3C). However, ASCs did not show any significant changes in cell cycle phases compared with control (untreated, Fig 3C). Cytotoxicity analysis by Annexin V/PI staining (Fig 3D) indicated a 19.3% of apoptosis in the CG5-PTX loaded ASC co-cultures, confirming the efficacy of PTX released by ASCs cells in the co-culture. Conditioned medium from PTX loaded ASCs determined a 22.9% of apoptosis in CG5-ASC co-cultures, demonstrating the high efficacy of PTX release from ASCs cells in the medium. To demonstrate that the cytotoxic effect was due to the release of PTX from ASCs in the medium and not to cell-cell direct contact, we performed MTT assay on CG5 cells cultured with the conditioned medium from PTX-primed ASC cultures. In the presence of 24h-conditioned medium from PTX (0.1–10μM)-loaded ASCs for 6h, CG5 proliferation was reduced after 7 days (IC50 = 0.029μM, p<0.01; Table 1). No effect was reported with conditioned medium from control unloaded ASCs or at time 0 of PTX release (data not shown).

### The therapeutic efficacy of PTX-primed ASCs *in vivo*

**Fig 4:**
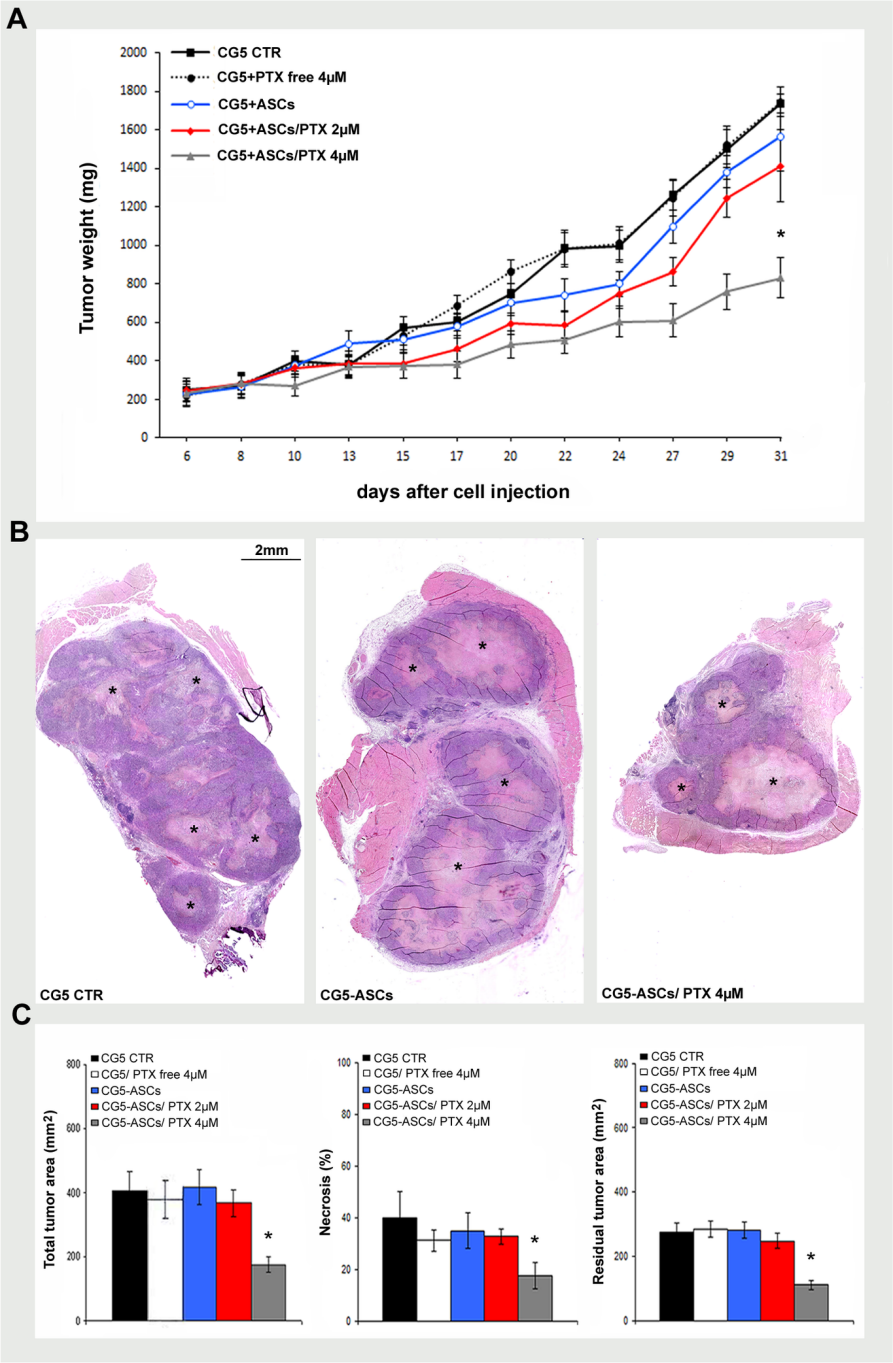
The therapeutic efficacy of PTX-primed ASCs on tumor growth *in vivo*. A) Tumor growth curve of CG5 cells (1x10^6^) co-injected i.m.in nude mice (5 mice/group) with ASCs (1x10^6^) unloaded or primed with PTX (2μM or 4μM for 6 hours), ratio of 1:1. Other control groups were CG5 alone or mixed with PTX free (4μM). B, C) Representative histological images and morphometric analysis of tumor xenografts showing total tumor area, percentage of necrosis, residual tumor area (mm^2^). Necrotic area, light pink eosinophilic area inside tumor mass (asterisk). *t*-test: *, p< 0.05.

**Fig 5:**
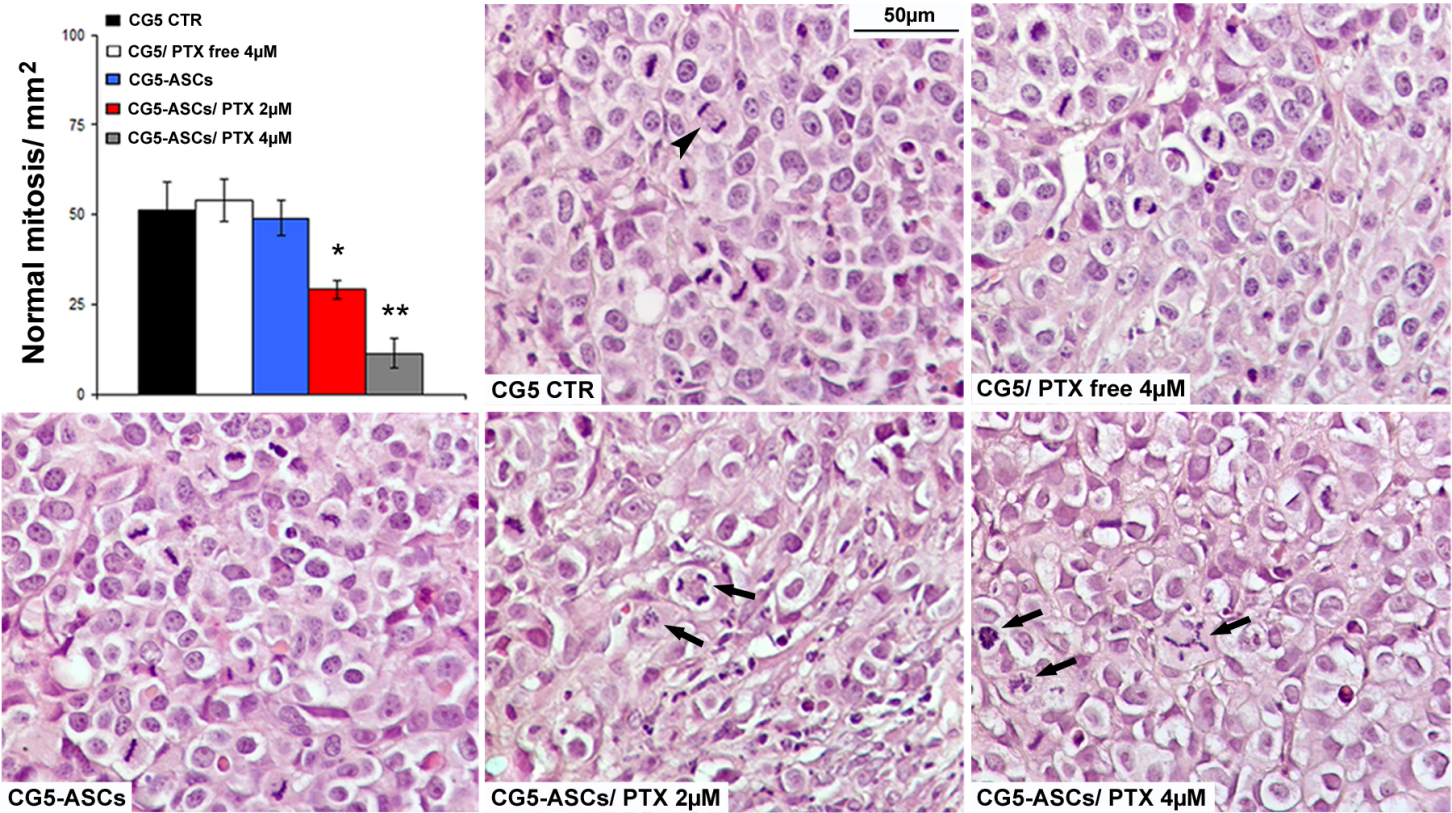
The effects of PTX-primed ASCs on tumor mitotic activity *in vivo*. Mitotic activity in high power field images of CG5 xenograft showing normal (bipolar, arrowhead) and aberrant mitosis (monoastral spindles and multipolar spindles, arrows). *t*-test: * and **, p< 0.05 and p< 0.01, respectively.

To translate the *in vitro* efficacy in an *in vivo* model, we assessed the therapeutic efficacy of PTX-primed ASCs in a mouse xenograft of breast cancer. A significant reduction of CG5 tumor xenografts (about 50%, maximum tumour volume 1,873 mm^3^) compared with controls (CG5 alone, CG5+unloaded ASCs or CG5+PTX free) was observed when breast cancer cells were injected, intramuscularly (i.m), mixed (ratio 1:1) with PTX-primed (4μM) ASCs (p< 0.05; Fig 4A). In addition, histological and morphometric analysis confirmed a reduction in tumor area as well as an increase of necrosis in CG5 tumor xenografts mixed with PTX-primed (4μM) ASCs (p<0.05, Fig 4B and 4C). The evidence of the PTX action was found in the mitotic activity observed in CG5 cells mixed with PTX-primed ASCs; in fact, a significant reduction of normal mitotic figures (bipolar mitosis) in favor of aberrant ones (monoastral spindles and multipolar spindles) with subsequent mitotic catastrophe was observed already starting from 2μM PTX (Fig 5). No toxicity or alterations were found in mice organs during necropsy (not shown). At lower concentrations of PTX, the injection (i.m.) with CG5 cells mixed with PTX (2μM)-primed ASCs (ratio 2:1), the experiment did not give the same results, rather an increasing trend of xenograft growth (Panel C in S1 Fig).

## Discussion

Breast cancer represents the main malignancy in women. Subjects after breast surgery, especially young patients, go to meet a poorer quality of life and psychological problems.

Regenerative surgery for breast reconstruction, such as autologous fat grafting, aims to minimize the aesthetic defects [47]. However, the absorption rate after fat grafting ranges from 25% to 80% after 1 year [13]. Actually, the finding that SVF/ASC enrichment enhances fat graft maintenance in breast reconstruction suggests a beneficial use in the routinely clinical practice [11,14]. In fact, the presence of ASCs has been supposed to be responsible for the survival and maintenance of the transplanted fat[14]. The differentiation potential of human ASCs provides anew prospective in the field of regenerative medicine [48]. The use of SVF/ASCs in several types of tissue reconstruction has raised a question regarding the safety of stem cell therapies in cancer patients, especially in the setting of reconstructive surgery after mastectomy[49]. However, the clinical application of ASCs needs further investigations. The relationship between adipose tissue and cancer cells was found out by Manabe *et al*. [50] that reported the implication of rat mature adipocytes (not preadipocytes) in the estrogen receptor-positive breast cancer cell growth *in vitro* [50]. Consequent works demonstrated that breast cancer growth and invasiveness were potentiated by ASCs [51,52]. Moreover, MCF-7 breast cancer cells, co-cultured with human ASCs, produced TGF-*β*1 and modulated the secretion of extracellular matrix [51]. In addition, the paracrine production of cytokines from ASCs seems to stimulate breast carcinoma cell growth [52,53] and angiogenesis [54]. Experimental data, from these studies, suggest that cancer invasiveness can be stimulated by ASCs through extracellular matrix remodeling and paracrine secretion. It was reported that ASCs differently communicate with active and quiescent breast cancer cells. In fact, the latter are rather independent and proliferate slowly [28,29]. To date, clinical trials and follow-up studies do not clarify about the increased risk of cancer recurrence or new onset after lipofilling [13,30–34]. In a study focused on patients previously diagnosed with breast intraepithelial neoplasia, the lipofilling patients did not display a significant higher risk of local recurrence than untreated subjects [31]. Similar data came from a clinical study conducted on 158 patients undergoing fat grafting after breast cancer surgery, and with 18 months of follow-up [33]. Some studies analyzed the local recurrence before and after lipotransfer in patients undergoing breast cancer surgery, and no significant difference was found [34]. Results from a small cohort of patients demonstrated that SVF, used for fat graft enrichment, do not increase the risk of breast cancer development [13]. However, in a study of 37 cases, the lipofilling group showed higher risk of local reappearance close to the lipofilling injection when the analysis was limited to breast intraepithelial neoplasia [32]. Some authors suggested c-Met expression, as a predictive factor to evaluate the risk of cancer recurrence following post-surgery autologous fat grafting in breast cancer patients [35]. Chemokines secreted by breast tumor cells may be capable of stimulating MSC migration and recruitment [40]. From this scenario of conflicting opinions, the role of ASCs in cancer progression is still debated [36].

Recently, some preclinical studies, based on MSC ability to home in the tumor microenvironment, suggest their use as candidates to delivery anti-cancer drugs [37–39]. Paclitaxel (PTX) is a widely used chemotherapic drug that acts as microtubule-stabilizing agent, inhibiting cancer cell mitosis [41]. It has been reported that MSCs and ASCs can uptake and subsequently slowly release PTX, inhibiting the proliferation of different cancer cells [39, 42–45]. this study, we demonstrated that ASCs can be loaded with PTX at high concentrations with not significant cytotoxic effects. Cell cycle analysis performed on total cell population, non-adherent and adherent PTX treated ASCs, showed a slight arrest in G2/M phase and increase of sub G0/G1 fraction, PTX treatment did not induce ASC apoptosis or necrosis as shown by Annexin V/PI assay on adherent cells. This finding was also confirmed by the high percentage of ASC survival with PTX treatment. Moreover, morphological and stem immunophenotypic features were unchanged.

On this point, some studies have been reported contradictory results; in fact, Huang et al. [54] reported that human ASCs showed no cytotoxic effects when engulfed in nanoparticles containing even 30μM of PTX, instead others demonstrated that PTX-loaded ASCs, starting from 0.01μM, showed a reduced viability and growth [55,56]. However, most of the works have highlighted the harmlessness of PTX exposure (up to 1μM and beyond) on ASCs [39,43,54,57].

The release of PTX by ASCs was confirmed by the cytotoxic effects of PTX-loaded ASC conditioned medium on CG5 cells. In fact, PTX-loaded ASC conditioned medium determined an evident arrest in G2/M phase and a strong increase of sub G0/G1 fraction. In addition, conditioned medium from PTX-loaded ASCs induced apoptosis and necrosis of CG5 and reduced their survival, showing a specific indirect cytotoxic effect for breast cancer cells. It is interesting to note that the cytotoxic effect of PTX free similar was similar to that of conditioned medium from PTX-loaded ASC cultures, strongly supporting an efficient ASC-mediated uptake and release of PTX. Moreover, a cytotoxic effect of PTX-loaded ASCs, in terms of apoptosis and clonogenicity, was observed in CG5/ASC co-cultures. Cell cycle analysis evidenced the different response of CG5 and ASCs to PTX treatment, with a strong arrest in G2/M phase and an increase of sub G0/G1 fraction for CG5 cells, and the minimal effect on ASCs. The small differences in terms of apoptosis of co-cultures compared to single populations were likely due to different culture conditions.

To verify *in vivo* the antitumor effects of PTX-loaded ASCs, we conducted experiments in a heterotopic model of breast cancer with CG5 xenografts with interesting results. Preliminary data using lower PTX concentrations (≤ 1μM) for ASC priming did not give the same cytotoxic and antitumor activity shown *in vitro*. These results are likely due to the different physiological conditions that occur *in vivo* compared with *in vitro* system. In fact, a significant reduction in tumor xenograft growth was observed, in a second experiment, when breast cancer cells were mixed with ASCs primed with 4μM of PTX, confirming the validity of CG5/ASC co-culture *in vitro* results. Histological and morphometric analysis of tumor xenografts confirmed the reduction of tumor area as well as the increase of tumor necrosis in the presence of PTX-primed ASCs, with no toxic effects or structural alterations in mice organs. PTX action was evident in the mitotic activity observed in CG5 cells mixed with PTX-primed ASCs; in fact, a significant reduction of normal mitotic figures (bipolar mitosis) in favor of aberrant ones, such as monoastral spindles and multipolar spindles, with subsequent mitotic catastrophe was observed already starting from 2μM PTX. This finding considers only GC5 activity because of their higher turnover and the specific sensitivity to PTX.

To date, just one study has reported the effectiveness of using bone marrow MSCs engulfed in PTX-loaded nanospheres in breast cancer growth inhibition by chemo-photothermal killing [45].The use of pre-loaded ASCs during post-surgical breast reconstruction may represent a potential new and easily accessible tool for adjuvant delivering anti-cancer drugs into the residual tumor microenvironment, so reducing the risk of local cancer recurrence but maintaining their regenerative properties. Nevertheless, further experimental *in vitro* and *in vivo* studies are needed to collect more evidences concerning the effect of PTX-loaded ASCs on tumor microenvironment. Moreover, the standardization and optimization of experimental conditions, as well as safety tests, are necessary to speculate a future clinical application of ASCs as a drug-delivery system in post-surgical breast reconstruction.

## Supporting information

S1 Fig Cell cycle and apoptosis of ASCs and CG5 with PTX treatments and preliminary experiment on the therapeutic efficacy of PTX-primed ASCs on tumor growth *in vivo*. A) Cell cycle analysis by flow cytometry of adherent and not adherent cells processed for PI staining. B) Cytofluorimetric bi-parametric analysis of the annexin V versus PI staining assay. The percentage reported in the annexin V^+^/PI^+^ region of each histogram represents the apoptotic cells. C) preliminary study was performed on nude mice (5 mice/group) injected intramuscularly (i.m.) with CG5 cells alone (3x10^6^) or mixed, at ratio of 2:1, with ASCs (1.5x10^6^) unloaded or primed with PTX (2μM) for 6 hours; tumor growth was reported. *t*-test.
(TIF)
