# Supplementary figures and images for: Adipose-derived stem cell-mediated paclitaxel delivery inhibits breast cancer growth
Source: PLoS One. 2018 Sep 7;13(9):e0203426. doi: 10.1371/journal.pone.0203426 (PMC6128546; doi:10.1371/journal.pone.0203426)

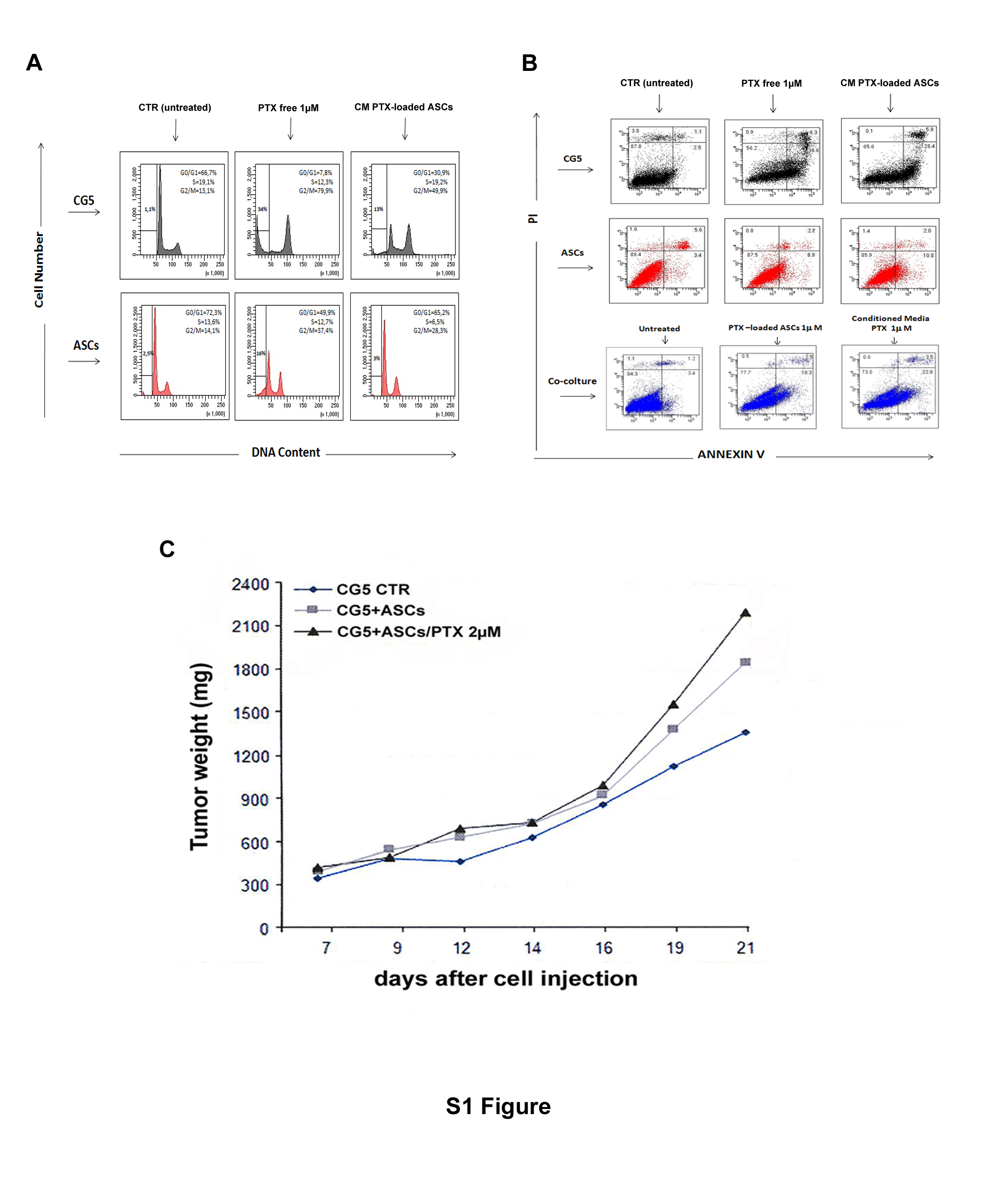

Supplement: S1 Fig — A) Cell cycle analysis by flow cytometry of adherent and not adherent cells processed for PI staining. B) Cytofluorimetric bi-parametric analysis of the annexin V versus PI staining assay. The percentage reported in the annexin V+/PI+ region of each histogram represents the apoptotic cells. C) preliminary study was performed on nude mice (5 mice/group) injected intramuscularly (i.m.) with CG5 cells alone (3x10^6^) or mixed, at ratio of 2:1, with ASCs (1.5x10^6^) unloaded or primed with PTX (2μM) for 6 hours; tumor growth was reported. t-test. (TIF) [file pone.0203426.s001.tif]
